# No Evidence of Chronic Infection in a Metagenomic Sequencing Study of the Keratoconus Corneal Epithelium

**DOI:** 10.3390/jcm13123399

**Published:** 2024-06-11

**Authors:** Pritpal Kaur, Loren Moon, Divya Srikumaran, Steven L. Salzberg, Jennifer Lu, Patricia J. Simner, Uri S. Soiberman

**Affiliations:** 1 The Wilmer Eye Institute, Johns Hopkins School of Medicine, Baltimore, MD 21287, USA; 2 Center for Computational Biology, Johns Hopkins University, Baltimore, MD 21218, USA; 3 Department of Biomedical Engineering, Johns Hopkins School of Medicine, Baltimore, MD 21218, USA; 4 Department of Computer Science, Johns Hopkins University, Baltimore, MD 21218, USA; 5 Department of Pathology, Johns Hopkins School of Medicine, Baltimore, MD 21287, USA

**Keywords:** keratoconus, metagenomic next-generation sequencing, ocular microbiota

## Abstract

**Objectives:** This study aims to assess the presence of pathogenic microorganisms in the corneal epithelial layer of keratoconus patients. **Methods:** DNA was extracted from corneal epithelial samples procured from ten individual keratoconus eyes and three healthy controls. Metagenomic next-generation sequencing (mNGS) was performed to detect ocular microbiota using an agnostic approach. **Results:** Metagenomic sequencing revealed a low microbial read count in corneal epithelial samples derived from both keratoconus eyes (average: 530) and controls (average: 622) without a statistically significant difference (*p* = 0.29). Proteobacteria were the predominant phylum in both keratoconus and control samples (relative abundance: 72% versus 79%, respectively). **Conclusions:** The overall low microbial read count and the lack of difference in the relative abundance of different microbial species between keratoconus and control samples do not support the hypothesis that a chronic corneal infection is implicated in the pathogenesis of keratoconus. These findings do not rule out the possibility that an acute infection may be involved in the disease process as an initiating event.

## 1. Introduction

The hallmark of keratoconus, a common corneal ectasia, is the presence of an irregular, cone-shaped cornea [1,2]. The prevalence rates of keratoconus vary between different reports but may be as high as 265 per 100,000 [3]. The mechanisms that lead to keratoconus remain poorly understood; both genetic factors (family history) and environmental factors (the habit of eye rubbing and nocturnal ocular compression) have been identified, and a possible role of pro-inflammatory cytokines has been suggested [4,5,6]. In addition to these factors, we hypothesized that keratoconus could also be caused by an infectious process because the disease onset is in childhood or early adolescence, a period in life when many infectious diseases are often acquired [3,7,8,9,10]. In addition, the geographical clustering of the disease may be suggestive of an infectious etiology because many infectious diseases tend to affect individuals who live in proximity to one another, and one of the most recent examples of that was COVID-19 [11,12,13].

Very few previous studies have studied the keratoconus ocular surface microbiome and have shown differences in the ocular microbiome of keratoconus patients when compared to controls [14,15]. Metagenomic analysis revealed that two genera, *Pelomonas* and *Ralstonia*, were unique in individuals with keratoconus [14]. While most ocular surface metagenomic data is derived from conjunctival swab samples, one recent study used epithelial samples and next-generation sequencing-based on 16S ribosomal RNA gene analysis, and it revealed that *Aquabacterium* was abundant in individuals with keratoconus even when compared to a negative control [15,16,17,18,19]. Since this study of corneal epithelium was performed on samples obtained outside North America, reflecting the microbiome of the local patient population, our study aimed to evaluate the presence of pathogens in keratoconus and control corneal epithelial samples derived from a patient population in Baltimore, MD, USA using metagenomic next-generation sequencing (mNGS).

## 2. Materials and Methods

### 2.1. Sample Preparation

The study was approved by the Johns Hopkins University School of Medicine Institutional Review Board, and all patients provided informed consent (IRB00112675). All study procedures adhered to the tenets of the Declaration of Helsinki. Corneal epithelial samples were obtained from keratoconus patients undergoing elective epithelium-off corneal collagen crosslinking and healthy control patients undergoing elective photorefractive keratectomy, in which keratoconus was ruled out. Both patient populations were treated with sterile proparacaine HCl 0.5% ophthalmic drops (Bausch & Lomb, Rochester, NY, USA) prior to epithelial debridement. Corneal removal was facilitated with a 30-s exposure to 20% ethyl alcohol followed by thorough irrigation with 15 mL of sterile balanced salt solution. Sterile cellulose sponges were used to remove the epithelium, and the samples were immediately placed in RNALater (ThermoFisher, Waltham, MA, USA) at 4 °C for 48 h, followed by storage at −80 °C. Topical anesthesia with sterile proparacaine HCl 0.5% ophthalmic drops and the application of 20% ethyl alcohol to the corneal surface does not affect the amount of microbial DNA in the corneal epithelium [20]. DNA extraction was performed using a DNeasy Blood & Tissue Kit (Qiagen, Hilden, Germany) in sterile conditions. DNA quality was determined with a NanoDrop ND-1000 Spectrophotometer (ThermoFisher, Waltham, MA, USA). RNAlater used for epithelium storage was processed as a negative control (NEC).

### 2.2. Metagenomic Next-Generation Sequencing

The sample was bead beaten and then underwent whole genome amplification (WGA) by REPLI-g WGA Kit (Qiagen, Hilden, Germany). Amplified products were diluted 1:100 in nuclease-free water and prepared for sequencing using the Nextera DNA Prep Kit (Illumina, San Diego, CA, USA) per manufacturer’s instruction, with the following addition: samples were quantified prior to library prep and normalized after library prep using a Qubit 3.0 fluorometer (ThermoFisher, Waltham, MA, USA) and TapeStation 4200 running HS DNA 5000 ScreenTapes (Agilent, Santa Clara, CA, USA). Samples were spiked with 1% phiX (Illumina, San Diego, CA, USA) and sequenced on the NextSeq 1000 (Illumina, Waltham, MA, USA) using the P2 100-cycle kit. Samples were accompanied by a blank RNAlater negative control.

### 2.3. Bioinformatics

For analysis of the sequencing reads, we first utilized the Illumina Experiment Manager (version 1.12.0) and the Illumina MiSeq Reporter (version 2.6.2.1) software for adapter trimming, followed by classification using KrakenUniq (database v.2016-01-13) as outlined previously [21]. KrakenUniq is a fast and accurate classification tool that includes unique k-mer counts to indicate coverage of classified reads across the genomic sequence [22]. For this analysis, we classified reads against a KrakenUniq database built in August 2020 that contains the NCBI RefSeq complete bacterial, archaeal, and viral genomes, the human GRCh38 reference genome, and common laboratory vectors. The Kraken results were then visualized through Pavian (version 1.0) [23]. Additionally, genomic coverage of positives was assessed through alignment using Bowtie2 (version 2.5.3) [24] for comparison purposes.

### 2.4. Statistical Analysis

GraphPad Prism 10 (GraphPad Software, La Jolla, CA, US) was used for additional statistical analysis. Mann-Whitney tests were used, and *p* ≤ 0.05 was considered statistically significant.

## 3. Results

Corneal epithelial samples were obtained from ten keratoconus eyes and three healthy controls, ranging in age from 15 to 25 years (see Table 1 for additional patient characteristics).

The mean number of raw reads was similar between the two groups (keratoconus: 34,846,225 ± 7,555,837, control 27,566,582 ± 1,801,193, *p* = 0.08, see Table 2). Most of the reads were successfully classified (keratoconus: 99.5% ± 0.11, control: 99.5% ± 0.11, *p* = 0.9), whereas in a negative control containing RNAlater but no tissue, approximately 80% of the reads were unclassified. Keratoconus and control samples had a very low number of microbial (bacterial, viral fungal, and protozoan) reads, and there was no difference between the groups (*p* = 0.94, Table 3).

**Figure 1:**
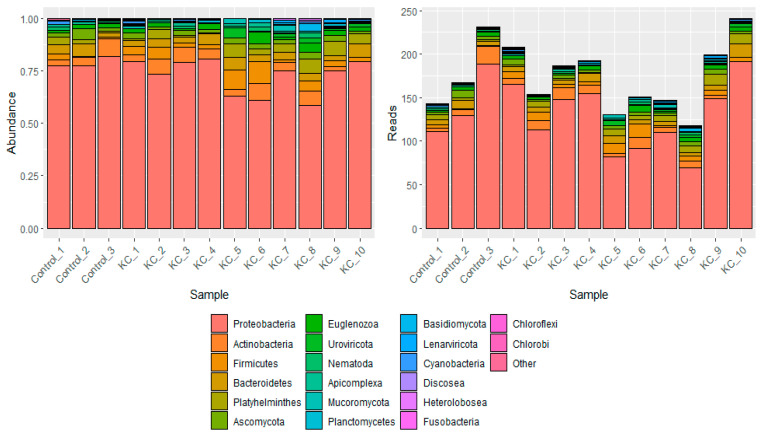
Phyla were identified by KrakenUniq across 13 corneal epithelial samples representing 3 healthy and 10 keratoconus samples. KrakenUniq was used to classify an average of 32.4 million reads per sample. The above plot represents the identified phyla excluding human reads. The top 20 phyla are represented, with the remaining reads grouped as “Other”. The left plot displays the relative abundance, while the right displays the actual read counts per phyla per sample. Control samples are healthy corneal samples, while KC samples represent the keratoconus samples.

Despite the high number of read counts generated per sample, analysis with KrakenUniq revealed that the majority of reads were of human source (≥99.3%), as expected given the nature of the samples (corneal epithelium). Microbial reads only accounted for a fraction of the total read count (on average 0.002%), with approximately 600 average microbial reads per sample. Of the microbial classified reads, Proteobacteria was the most abundant Phyla, followed by Actinobacteria and Firmicutes (Figure 1).

**Figure 2:**
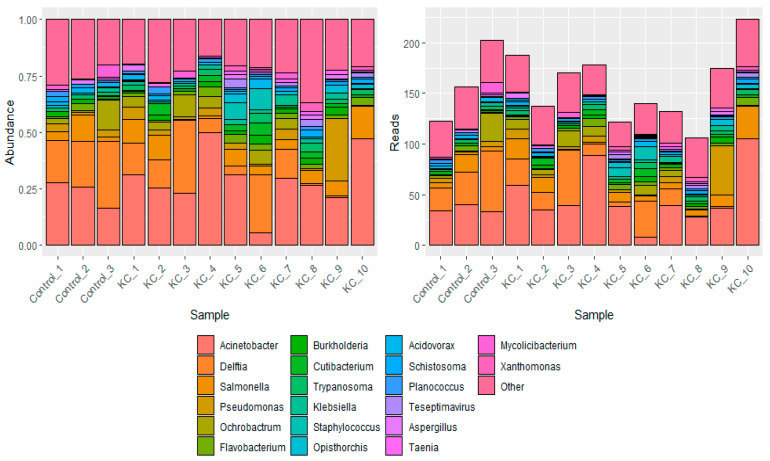
Genera identified per corneal epithelial sample by KrakenUniq classification. This plot displays the identified genera across the 3 healthy “Control” samples and the 10 keratoconus samples. Human reads were excluded from this plot for clarity. The left plot displays the relative abundance of the genera across the 13 samples, while the right plot displays the exact read counts per genus per sample.

Further analysis by KrakenUniq and Pavian revealed slight differences in classification at the genus level. For example, the control samples had an average of 38 Delftia reads per sample, while the same genus had an average of 17 reads per keratoconus sample, yielding a 2.3× difference between the two (*p* = 0.09, Figure 2). Additionally, KrakenUniq revealed a higher number of Acinetobacter, Salmonella, and Pseudomonas reads in the keratoconus samples as compared to the control samples (*p*-value > 0.05 for all comparisons, Figure 2). However, these genera were much more abundant in negative control, suggesting contamination from the experimental reagents. The overall number of microbial reads was extremely low compared to the total number of sequenced reads, with <100 reads classified per genus per sample. The low read count precluded these results from being clinically meaningful.

## 4. Discussion

Our metagenomic study shows no evidence to suggest a chronic infection in the keratoconus corneal epithelium. This finding refutes our initial hypothesis that an initial infectious insult to the corneal epithelium may lead to a cascade of events that result in a final keratoconus phenotype. It remains unclear whether metagenomic sequencing is sensitive enough to detect remote infection; however, other studies that used polymerase chain reaction (PCR) have successfully detected Herpes family viruses (including HSV1, HSV2, VZV, and CMV) even in quiet asymptomatic corneas. Since these infections are often acquired during childhood and adolescence, they suggest that even latent infections can be detected with PCR and metagenomic sequencing [25,26,27,28]. However, we identified minor differences in our patient samples that were not significant. Notably, our negative controls exhibited a higher abundance of reads for all identified bacteria. This observation suggests that detected reads may be attributed to nucleic acid contamination in the sequencing reagents.

Other groups have also studied the ocular microbiome in keratoconus, as it is well known that conjunctival disease, especially atopy, is associated with keratoconus, and there have been previous associations between ocular surface disease and altered microbiome [16]. In a study conducted on 38 keratoconus adult patients and 167 healthy controls, 16S rRNA sequencing was performed on conjunctival swab specimens to reveal significant differences in microbiota; *Pelomonas* and *Ralstonia* were more abundant in the keratoconus population. Although these genera are often erroneously detected due to kit reagent contamination, this study used a negative control. Our study did not detect these genera in our samples, yet we analyzed corneal epithelial samples rather than conjunctival swab samples of the ocular surface.

In another metagenomic study performed on corneal epithelial samples, keratoconus samples had a higher relative abundance of twenty different genera, with *Aquabacterium* being the predominant genus [15]. In that study, *Aquabacterium* was present in all samples, and the average abundance in keratoconus epithelium was approximately 11%; however, this finding was not statistically significant when compared to myopic controls. In our study, this specific genus was undetected in both healthy and keratoconus samples. Since microbiomes can vary by location, we attribute this difference between studies to geographical variability.

Our study is limited by its scope: we investigated the central corneal epithelium only, whereas a potential pathogen may favor another anatomical location, such as the limbus or the stromal keratocytes. Our study sample size is small but was deemed sufficient for this comparison; of note, there are no clear guidelines for power calculations in the realm of metagenomic sequencing studies. Our study hypothesis was that an infectious insult to the corneal epithelium may trigger a series of events that lead to keratocyte death and collagen loss. However, it is possible that this potential pathogen is cleared at some point after the onset of the infection and is, therefore, undetectable when the samples are sequenced years later. Since patient age has a great impact on the results of ocular microbiome metagenomic sequencing, we suggest that future studies will include samples derived from younger keratoconus patients with earlier disease, who may exhibit different results [20]. Our negative control sample yielded a higher microbial read count than the other tissue samples, underlining the low number of microbial reads from the corneal epithelium. However, most of the reads from the negative control were unclassified, whereas most of the reads from tissue samples were human. Since the sequencing study is limited by its total number of reads and because our study was performed on corneal epithelial samples (not conjunctival swabs), the increased number of human reads may have reduced the potential number of detectable microbial reads [29]. Finally, all of the genera identified in our samples were present in the negative control, further suggesting that their presence does not have any clinical significance.

In conclusion, our metagenomic study of corneal epithelium did not detect a specific pathogen associated with keratoconus.

## Figures and Tables

**Table 1:** Patient characteristics.

Patient	Gender	Age (Y)	Ethnicity	Race
Control 1	M	21	Not Hispanic	White
Control 2	M	21	Not Hispanic	White
Control 3	F	25	Not Hispanic	White
KC-1	M	19	Did not disclose	Did not disclose
KC-2	M	15	Not Hispanic	Black
KC-3	M	20	Not Hispanic	Unknown
KC-4	M	26	Not Hispanic	Black
KC-5	M	21	Not Hispanic	Black
KC-6	F	21	Not Hispanic	Asian
KC-7	M	18	Not Hispanic	Unknown
KC-8	M	15	Not Hispanic	White
KC-9	M	18	Not Hispanic	Black
KC-10	M	23	Not Hispanic	White

KC: keratoconus; M: male; F: female.

**Table 2:** Sequencing results: number of raw, classified, unclassified, and human reads.

Sample	Raw Read Count	Classified Reads	Classified %	Unclassified Reads	Unclassified %	Human %
Control 1	26,757,195	26,585,004	99.4%	172,191	0.6%	99.3%
Control 2	29,630,482	29,466,686	99.4%	163,796	0.6%	99.4%
Control 3	26,312,069	26,198,852	99.6%	113,217	0.4%	99.6%
KC 1	30,645,744	30,437,780	99.3%	207,964	0.7%	99.3%
KC 2	29,073,829	28,889,589	99.4%	184,240	0.6%	99.4%
KC 3	23,346,495	23,212,141	99.4%	134,354	0.6%	99.4%
KC 4	32,998,728	32,870,823	99.6%	127,905	0.4%	99.6%
KC 5	29,986,363	29,853,060	99.6%	133,303	0.4%	99.5%
KC 6	30,944,636	30,815,579	99.6%	129,057	0.4%	99.6%
KC 7	42,074,977	41,792,526	99.3%	282,451	0.7%	99.3%
KC 8	47,616,326	47,356,357	99.5%	259,969	0.5%	99.4%
KC 9	41,005,109	40,781,373	99.5%	223,736	0.5%	99.4%
KC 10	40,770,043	40,545,853	99.5%	224,190	0.5%	99.4%
NEC	22,425,709	4,752,704	21.2%	17,673,005	78.8%	11.3%

KC: keratoconus; NEC: negative control.

**Table 3:** Number of total microbial reads and their distribution.

Sample	T.M.R.	M.R. %	Bacteria	Archaea	Viruses	Fungi
Control 1	497	0.002%	130	0	1	3
Control 2	526	0.002%	148	0	0	11
Control 3	567	0.002%	217	0	1	5
KC 1	593	0.002%	191	0	1	9
KC 2	573	0.002%	139	0	1	2
KC 3	496	0.002%	173	0	0	5
KC 4	576	0.002%	181	0	0	5
KC 5	452	0.002%	106	0	6	7
KC 6	503	0.002%	128	0	1	4
KC 7	672	0.002%	125	0	4	8
KC 8	786	0.002%	88	0	3	8
KC 9	790	0.002%	167	0	4	8
KC 10	780	0.002%	217	0	5	3
NEC	2,164,853	45.550%	2,145,997	502	2369	4603

T.M.R.: total microbial reads; M.R.: microbial reads; KC: keratoconus; NEC: negative control.

## Data Availability

All data are contained within the manuscript.
